# Automatic literature screening using the PAJO deep-learning model for clinical practice guidelines

**DOI:** 10.1186/s12911-023-02328-8

**Published:** 2023-11-03

**Authors:** Yucong Lin, Jia Li, Huan Xiao, Lujie Zheng, Ying Xiao, Hong Song, Jingfan Fan, Deqiang Xiao, Danni Ai, Tianyu Fu, Feifei Wang, Han Lv, Jian Yang

**Affiliations:** 1 School of Medical Technology, Beijing Institute of Technology, Beijing, 100081 China; 2 Department of Radiology, Beijing Friendship Hospital, Capital Medical University, Beijing, 100050 China; 3 School of Statistics, Renmin University of China, Beijing, 100872 China; 4 School of Computer Science & Technology, Beijing Institute of Technology, Beijing, 100081 China; 5 School of Automation, Beijing Institute of Technology, Beijing, 100081 China; 6 Beijing Engineering Research Center of Mixed Reality and Advanced Display, School of Optics and Photonics, Beijing Institute of Technology, Beijing, 100081 China; 7 Center for Applied Statistics, Renmin University of China, Beijing, 100872 China

**Keywords:** Clinical practice guideline, Curation, Deep learning, Natural language processing

## Abstract

**Background:**

Clinical practice guidelines (CPGs) are designed to assist doctors in clinical decision making. High-quality research articles are important for the development of good CPGs. Commonly used manual screening processes are time-consuming and labor-intensive. Artificial intelligence (AI)-based techniques have been widely used to analyze unstructured data, including texts and images. Currently, there are no effective/efficient AI-based systems for screening literature. Therefore, developing an effective method for automatic literature screening can provide significant advantages.

**Methods:**

Using advanced AI techniques, we propose the Paper title, Abstract, and Journal (PAJO) model, which treats article screening as a classification problem. For training, articles appearing in the current CPGs are treated as positive samples. The others are treated as negative samples. Then, the features of the texts (e.g., titles and abstracts) and journal characteristics are fully utilized by the PAJO model using the pretrained bidirectional-encoder-representations-from-transformers (BERT) model. The resulting text and journal encoders, along with the attention mechanism, are integrated in the PAJO model to complete the task.

**Results:**

We collected 89,940 articles from PubMed to construct a dataset related to neck pain. Extensive experiments show that the PAJO model surpasses the state-of-the-art baseline by 1.91% (F1 score) and 2.25% (area under the receiver operating characteristic curve). Its prediction performance was also evaluated with respect to subject-matter experts, proving that PAJO can successfully screen high-quality articles.

**Conclusions:**

The PAJO model provides an effective solution for automatic literature screening. It can screen high-quality articles on neck pain and significantly improve the efficiency of CPG development. The methodology of PAJO can also be easily extended to other diseases for literature screening.

## Background

Clinical practice guidelines (CPGs) are curated collections of the best practices used to guide, optimize, and establish norms for clinical practice and are thus essential to clinicians, administrators, the public, and program managers [1]. CPGs are built using materials with quality evidence [2], which implies that clear, explicit, and unbiased information is selected. Hence, CPGs require frequent systematic reviews to ensure their curation and reduce the risk of medical malpractice.

Scholarly published articles are the key source of critical evidence that feeds CPGs, leading to a regular need for screening the most recent evidence based on research topics. However, the number of articles is witnessing an exponentially growth, it is reported that over 120 million papers have been published so far [3]. This large volume of papers brings enormous challenges for curation. Besides, curator selection is restricted to top field experts, making curation scheduling a tough, time-consuming task. Undesirable selective bias and human mistakes occur. In this regard, an automated curating tool for overall reviewing and assessing the quality of domain-related publications could be of use to CPG creators.

We first assume that there are clearly identifiable features that delineate high-quality articles from the rest. Neural networks have made vast improvements in the identification and assessment of text features. Several have already been adapted for medical text analyses. Advanced natural language processing (NLP) methods are now being used in many fields for literature screening.

Presently, traditional classifiers such as, random forest and support vector machine (SVM) models are effectively applied to simple medical text-processing tasks. For example, a term frequency–inverse document frequency (TF-IDF) feature extraction technique was developed with a naïve Bayes classifier that automatically screens for medical guidelines [4]. The SVM classifier was used to screen medical articles [5]. Compared with the traditional TF-IDF feature engineering strategy, the deep learning method was also applied and performed more effective than the TF-IDF method [6]. An ensembled method based on classical machine learning and deep learning approaches was further adopted, which improving the performance of the single best model on small datasets [7]. These traditional models facilitate comprehensive information mining by ranking features of the texts, leading to interpretable results.

With the development of deep-learning techniques, more complex and advanced methods are now available improving the performance of text mining [8]. An attention-based convolutional neural network (CNN) was adopted for medical code prediction [9]; this first aggregated information from a document using a CNN, and it then applied an attention mechanism to select the most relevant segments, making accurate selections from thousands of possible lines of code. The CNN and long short-term memory (LSTM) models was further combined, where the CNN was used to extract word-level semantic features, and the LSTM was used to extract timing characteristics [10]. A composite index test algorithm for literature screening was proposed in [11]. Various bidirectional-encoder-representations-from-transformers (BERT) methods have been adapted for text processing problems, including “A Lite” BERT [12], “scientific” BERT [13], and “biomedical” BERT [14]. For example, Moen et al. [15] combined the prediction results of eight models, including a BERT and a bidirectional LSTM (BiLSTM), to determine an article’s relevancy. A Knowledge Language Model (K-LM) model was developed for knowledge injection based on Generative Pre-trained Transformer 2 (GPT-2) and BERT, which improved the performance relative to classical machine learning methods [16]. As demonstrated by numerous experiments, BERT models do an excellent job of “understanding” text following sufficient model training. Additionally, they can be flexibly combined with ancillary network structures, depending on the task at hand. Compared with traditional deep-learning NLP models, the BERT models are the best.

This study seeks to provide the capability to quickly locate and classify high-quality medical studies. As a starting point, we focus the scope on the diagnosis and treatment of neck pain. To this end, we construct a dataset of candidate articles from PubMed. Those cited by the extant CPG, as well as systemic scholarly reviews, are regarded as positive samples for model training; all others are treated as negative. Using this, we provide a binary text classification problem for a BERT NLP model. Various attributes from the textual information found in the articles, alongside selected journal characteristics, are used for feature extraction and analysis. The resulting novel Paper title, Abstract, and JOurnal (PAJO) model, which is based on the pretrained PubMedBERT model, was applied to neck-pain medical article screening for generating CPG. Compared with the best baseline, the Text-based Recurrent Convolutional Neural Network (TextRCNN), the PAJO model achieves 1.91% improvement in the F1-score and 2.25% in the area under the receiver operating characteristic curve. This research article presents the following contributions of our study:

- We developed a novel PubMedBERT-based PAJO deep-learning neural network, which mines the textual information of articles and the journal characteristics for their feature information to CPG article screening.
- We designed a general framework for automatic medical literature screening that includes data collection, feature extraction, model-building, and performance evaluation.
- Taking neck-pain as a case study, we demonstrated that the proposed PAJO model can accurately screen a greater number of high-quality neck-pain articles for curating the related CPGs, when compared to several state-of-the-art methods.

The remainder of this paper is organized as follows. Methods section explains the PAJO methodology. Results section presents our experimental results compared with several state-of-the-art methods, and Discussion section presents our ablation study. Finally, Conclusions section concludes this paper.

## Methods

### Dataset construction

**Fig. 1:**
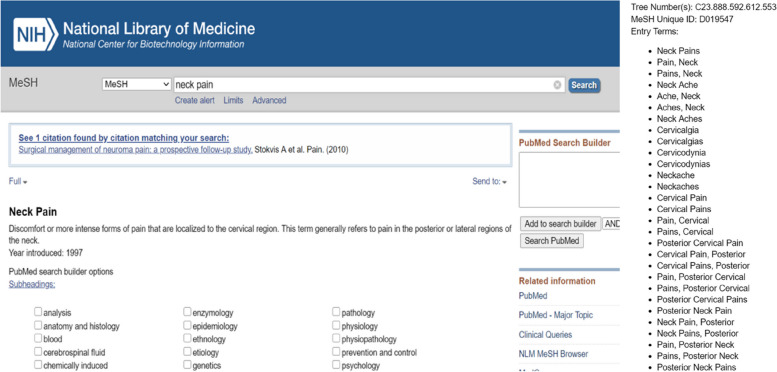
Mesh search terms related to “neck pain.” The left panel displays the retrieval interface and search term of “neck pain,” and the right panel lists the entries found, which can be selected for further screening

Dataset construction is the first step in deep-learning model training. To empower our model to automatically screen high-quality, scientifically rigorous articles related to neck pain, we queried the PubMed^1^ database in Dec. 2021, which stores more than 20 M biomedical articles. PubMed’s MeSH tool is a powerful query method that allows researchers to search for various combinations of keywords and phrases expressed as Boolean relationships. Our “neck pain” query resulted in 41 entry terms, as shown in Fig. 1.

These 41 terms were regarded as our initial set of keywords and phrases related to “neck pain.” To reduce the size of this set, we conducted a literature survey on neck pain and found the most used keywords. We verified our selection with experts from the field. We then narrowed the keyword and phrase combinations to “back pain,” “pain back,” “neck pain,” “pain neck,” and “cervical pain.” Using these five entries, we undertook our secondary search.

We used MeSH to perform a fuzzy search on the entire PubMed database, with our five keywords and phrases targeting the fields *title*, *abstract*, and *publisher*. We then matched the five key phrases with the collection of titles and abstracts using a fuzzy retrieval strategy. For example, in any given article, if the two words comprising a key phrase are separated by no more than three additional words, this meets our matching rule; the title “Consensus practice guidelines on interventions for cervical spine joint pain from a multispecialty international working group” contains the words “cervical” and “pain,” separated two other words, “spine joint.” This meets the “cervical pain” keyword and phrase criteria. Hence, this title is included in the final dataset.

**Fig. 2:**
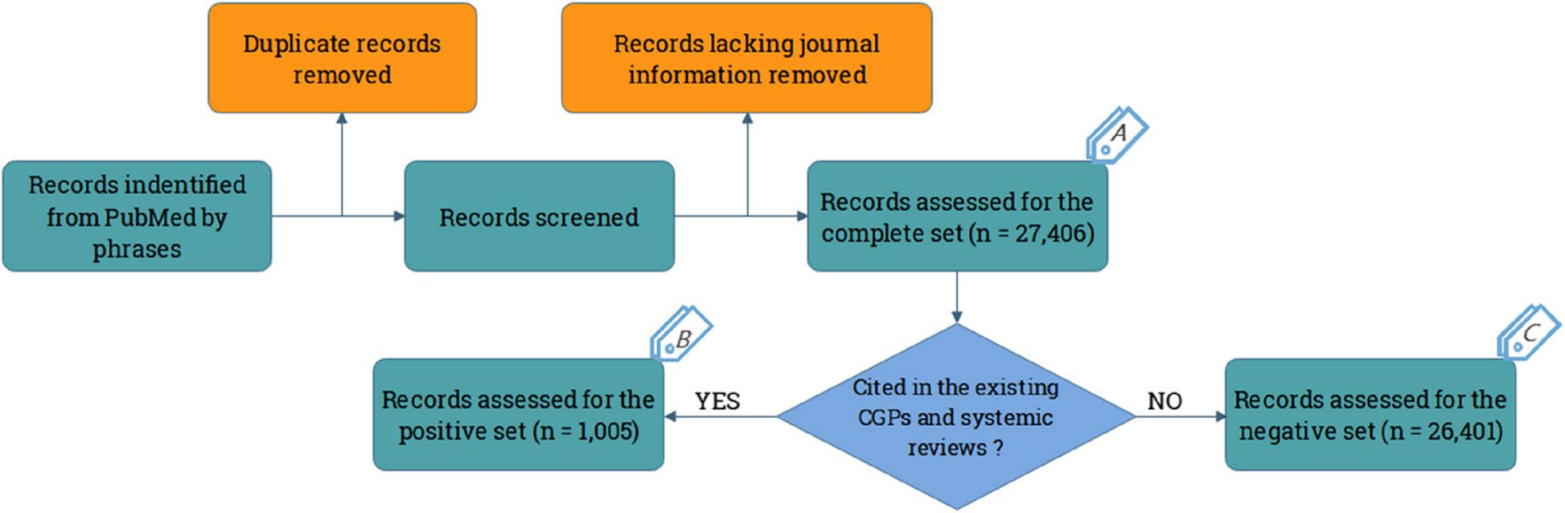
Dataset construction process. We first applied our keyword and phrase retrieval matching rule to all PubMed articles. We then performed deduplication and removed records for which the associated journal information was unavailable. Finally, we classified Set A as the complete collection of 27,406 samples. Set B was classified with 1,005 articles that were cited in the CGPs and systematic reviews (positive samples). Finally, Set C contained the 26,401 negative samples

Using this method, 89,940 articles were retained. Data preprocessing was then performed as follows. First, the duplicate articles were removed. Second, noting that the publishing journal identification provides valuable information about the article’s quality, articles lacking the associated journal information were discarded. A total of 27,406 articles remained to comprise Set A (the complete collection). From this set, 1,005 articles were cited in the existing CGPs and systemic reviews for neck pain; thus, they were deemed the most important for our task. These articles comprised Set B (positive samples). The remainder (26,401) was regarded as Set C (negative samples). Obviously, $\text{A}=\text{B}\cup \text{C}$. Hence, in this study, we used Sets B and C for positive and negative model training, respectively. The specific dataset construction process is illustrated in Fig. 2.

### Handling category imbalances

Given that our ratio of positive to negative samples was $1:28.3\approx 0.035$, we faced an extremely imbalanced case that would not result in good model training. Hence, we applied a focal loss function to dichotomize the unbalanced data [17]. For each sample *i*, ${Y}_{i}=1$if it is positive; otherwise, ${Y}_{i}=0$(negative). Furthermore, ${p}_{i}=P({Y}_{i}=1)$, which reflects the probability that a classification model predicts a positive sample. Let ${\widetilde{p}}_{i}={p}_{i}$ if ${Y}_{i}=1$, and ${\widetilde{p}}_{i}=1-{p}_{i}$ if ${Y}_{i}=0$. For sample *i*, the focal loss is defined as follows:

1 $$FL\left(i\right) = -{\tilde\alpha }{(1-{\tilde{p}}_{i})}^{\gamma}\,\text{log}\left({\tilde{p}}_{i}\right).$$

Here, two hyperparameters were used in the focal loss. Hyperparameter $\gamma$ is the exponent of the modulating factor, which is usually a positive integer. This reduces the contributions of easily separable samples and increases the hard-separable proportion for balancing. Hyperparameter ${\tilde\alpha }={\upalpha }$ if ${Y}_{i}=1$, and ${\tilde\alpha }=1-{\upalpha }$ if ${Y}_{i}=0$, giving us a weighting factor in [0, 1], which is used to adjust the ratio between positive and negative sample losses. To find the appropriate values of ${\upgamma }$ and ${\upalpha }$, we first set a varying range for each hyperparameter based on our preliminary analysis. Then we used grid search to find the optimal values. We found the best hyperparameter values were ${\upgamma }=2$ and ${\upalpha }=0.8$. We also applied a downsampling technique to Set C (negative) to supplementarily balance the data for subsequent model training, validation, and testing. Downsampling is a widely applied technique to balance the sample sizes in datasets. In this work, Set C has a much larger sample size than Set B. To balance these two datasets, we need to select a subset from Set C. To this end, we first assign each negative sample in Set C with an equal sampling probability. Then we randomly selected 2,345 samples from Sec C using the technique of sampling without replacement. The final ratio of positive to negative samples was 3:7.

### Feature extraction

When contemplating feature extraction, we were burdened with creating a deep-learning model from scratch, including selecting and testing its subcomponents. Fortunately, because our research field is closely related to biomedicine, we noted that a pretrained PubMedBERT NLP already exists in that field [18]. Different from other BERT-type models, which are trained on millions of articles related to a wide range of topics, PubMedBERT is pre-trained from scratch on biomedical research literature. By training PubMedBERT with our information collected from PubMed, we could directly obtain relevant predictions based on “neck pain.”

To identify journal features, we leveraged the following pre-existing attributes:

- Journal Impact Factor (IF) [19]. This feature reflects the “influence” of academic journals in terms of their average annual citations in new articles. To account for IF value fluctuations over time, we considered them annually from 2015 to 2021.
- CiteScore (CS) [20]. This feature reflects an Elsevier metric launched in 2016 that conveys the annual citations per article per journal compiled from the Scopus database over the previous four years.
- Scientific Journal Ranking (SJR) [21]. This feature reflects both the number and quality of citations and weighs those of prestigious journals.
- Source-Normalized Impact per Paper (SNIP) [22]. This feature reflects another Elsevier measurement issued in 2012 that uses the Scopus database. It is the reference weight based on the total number of citations in a subject area. Therefore, a citation is assigned a higher value if it is cited in disciplines outside its domain. SNIP also corrects for differences in journal citation behaviors in different subject areas.
- The Science Citation Index or Journal Citation Reports Divisions of the Chemical Abstracting Service. This feature reflects 14 major disciplines, and in each, journals are ranked according to their impact factors: Zone 1 (top 5%), Zone 2 (top 5–20%), Zone 3 (top 20–50%), and Zone 4 (the remainder).
- The H-Index [23]. This feature reflects the productivity and impact of a researcher or journal and is calculated based on the number of articles published by a journal and the number of times an article is cited. A journal with *n* articles cited at least *n* times each has an H-index of *n*.

### The PAJO classification model

**Fig. 3:**
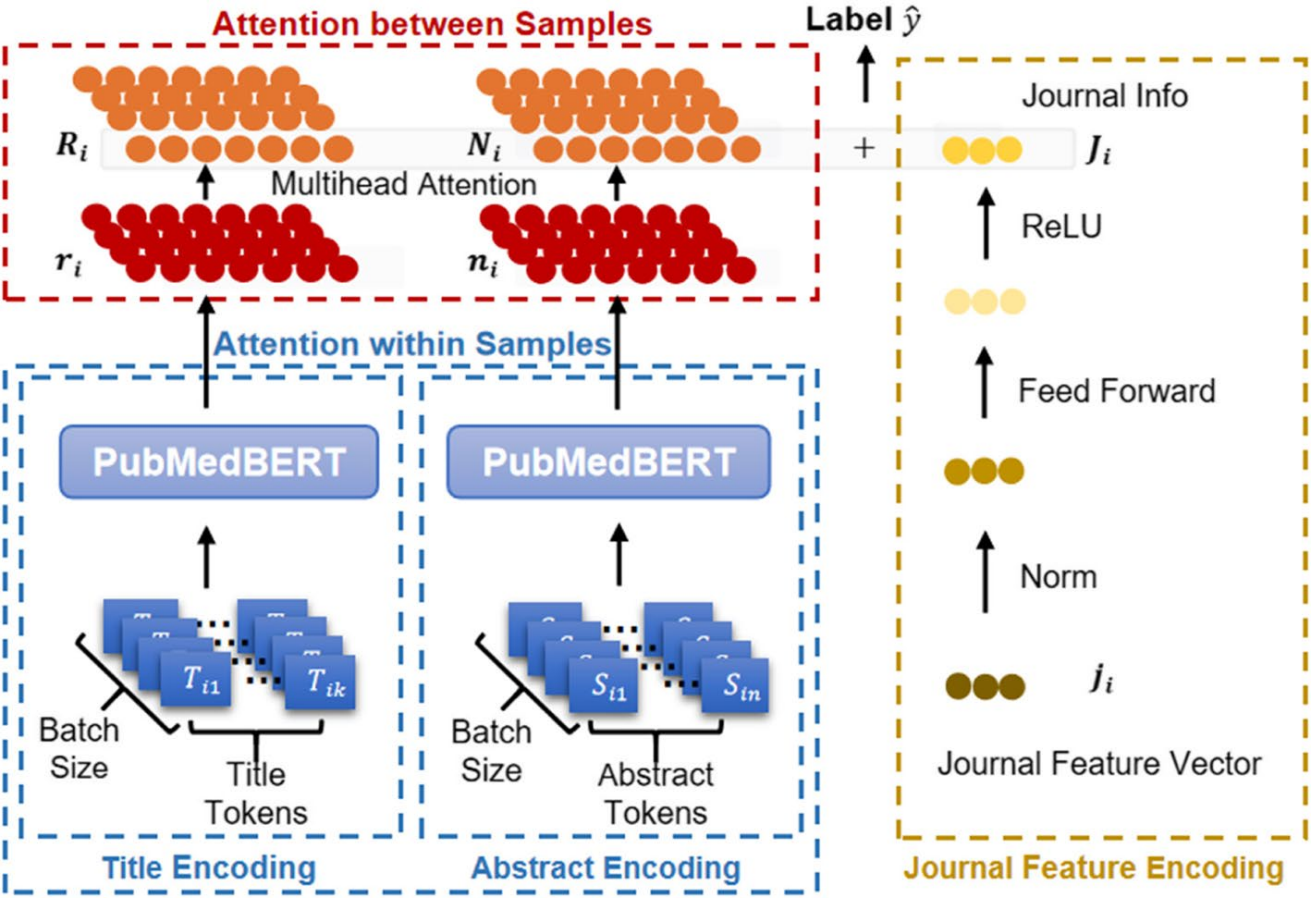
PAJO network architecture. Each article’s raw title and abstract are fed into the PubMedBERT text encoder for conversion to embedding vectors. The vectors are passed to an attention encoder for weight sample representation. The original journal features are normalized and passed to a feed-forward layer with a rectified linear unit (ReLU) activation function. The obtained text and journal features are concatenated to obtain the overall feature representation of an article. Finally, the feature representation is passed to a feed-forward layer with a SoftMax function to predict the article’s label

Our PAJO model has three modules: an in-sample text feature encoder that converts title and abstract strings to embedding vectors, an attention encoder that converts inter-sample text feature vectors from single samples into weighted representations between samples, and a journal feature encoder that extracts the journal features listed in Feature extraction section. PAJO’s network architecture is illustrated in Fig. 3.

The text encoder module focuses on in-sample text feature representations. For each sample, *i*, we use the PubMedBERT tokenizer to tokenize the title’s text as sequence ${T}_{i}$. Similarly, the abstract is tokenized as ${S}_{i}$. Subsequently, both ${T}_{i}$ and ${S}_{i}$ are fed to PubMedBERT for encoding, where an attention mechanism allows the embedding vector corresponding to each word to incorporate the information of all words in the text. In the last hidden layer, the embedding associated with the reserved [CLS] token is used for downstream classification tasks [24]. We define ${r}_{i}$ as the in-sample vector representation of ${T}_{i}$ and ${n}_{i}$ as the inter-sample vector representation of ${S}_{i}$, which is represented as follows:

2 $${r}_{i} = \text{P}\text{u}\text{b}\text{M}\text{e}\text{d}\text{B}\text{E}\text{R}\text{T}\left({T}_{i}\right),$$

3 $${n}_{i} = \text{P}\text{u}\text{b}\text{M}\text{e}\text{d}\text{B}\text{E}\text{R}\text{T}\left({S}_{i}\right).$$

We define *d* as the embedding size, where ${r}_{i}, {n}_{i}\in {\text{R}}^{d}.$ The word embedding and encoding layers in PubMedBERT are fine-tuned by our dataset in the model training process. Note that ${r}_{i}$and ${n}_{i}$ are associated with [CLS] tokens and contain information about the entire text.

The second PAJO module is an attention encoder that focuses on inter-sample text feature representations. The title vector from each article is recoded as a weighted title vector between articles using the attention mechanism, which forces the model to learn from the subtle gaps in title and abstract representations across multiple articles. Define the batch size to be *s*, which is the number of samples analyzed in each epoch. Then $r\;=\;\left[r_1,\;r_2,\;\dots,\;r_s\right]^{\mathrm T}$ denotes the in-sample title vector representation used for the entire batch. We denote $\text{Q},\text{K},\text{V}\in {\text{R}}^{d}\times {\text{R}}^{d}$ as the corresponding trained query, key, and value matrices, respectively. We then multiply *r* by training matrices ${\text{W}}^{\text{Q}}$, ${\text{W}}^{\text{K}}$, and ${\text{W}}^{\text{V}}$ to obtain Q, K, and V, respectively. Hence, $\text{Q} =\text{r}{\text{W}}^{\text{Q}}$, $\text{K} =\text{r}{\text{W}}^{\text{K}}$, and $\text{V} =\text{r}{\text{W}}^{\text{V}}$. We define ${\alpha }_{i}$ as the computed weight vector of Sample *i*, which is represented by other in-sample text vectors from the same batch. The title text feature, ${\text{R}}_{i}$, is formulated as follows:

4 $${\alpha }_{i} = \text{S}\text{o}\text{f}\text{t}\text{m}\text{a}\text{x}\left({q}_{i}{\text{K}}^{\text{T}}\right),$$

5 $${\text{R}}_{i} = {\alpha }_{i}\text{V} .$$

Ideally, when applying the attention mechanism to calculate the weights of vectors between samples, every sample in the dataset should be considered. However, owing to limited computing resources, we created sample sets from each batch. Thus, all text vectors are weighted in the same batch to obtain the inter-sample representation of each vector.

The third PAJO module is the journal feature encoder, with which the categorical features based on the journal characteristics are transformed into dummy variables. Continuous features are normalized, and all categorical and continuous features are concatenated to obtain the full journal feature vector, ${j}_{i}$ for sample *i*. This is then submitted to a feed-forward layer for the linear combination of different journal features. Finally, the output features are fed into the ReLU activation function, which is commonly used in deep neural networks.

For sample *i*, we then obtain its intersample title text feature${R}_{i}$, intersample abstract text feature${ N}_{i}$, and enhanced journal feature${J}_{i}$. By concatenating these, we obtain the overall feature, ${X}_{i}$, for final classification, where ${X}_{i} = contact({R}_{i},{N}_{i},{J}_{i})$. For a batch with *s* samples, $X\;=\;\left[X_1,\;X_2,\;\dots,\;X_s\right]$, and its predicted labels are $\widehat{Y}$. Thus, in the fully connected layer, we have

6 $$\widehat{Y}=\text{S}\text{o}\text{f}\text{t}\text{m}\text{a}\text{x}\left(WX+b\right),$$

where $W\in {\text{R}}^{\stackrel{\sim}{d}}{\times \text{R}}^{\stackrel{\sim}{d}}$, and $\stackrel{\sim}{\text{d}}$is twice the embedding size plus the journal feature size. Finally, we summarize the implementation of PAJO in Algorithm 1.

**Figure:**
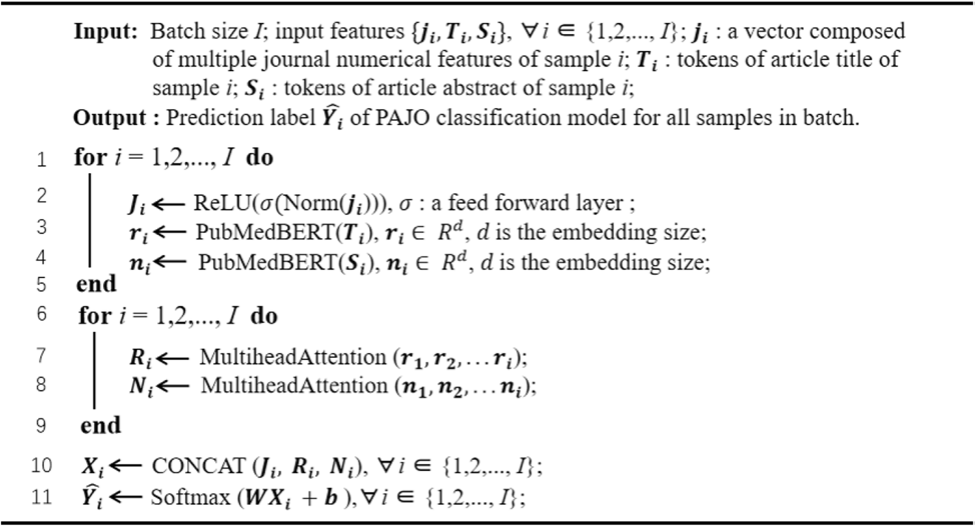
**Algorithm 1:** Label prediction process of PAJO classification model

## Results

### Models and Metrics

We conducted a series of experiments to investigate the classification performance of our proposed PAJO model. The corresponding data and codes are available at https://github.com/xh621/PAJO-Deep-Learning-Model. For comparison purposes, we considered several competitors, including CNNs, recurrent neural networks (RNNs), other attention mechanisms, and pretrained language models. The text and journal feature inputs are similar for all models.

- Random Forest [25]. With this model, the title, abstract, and journal features are concatenated and fed to a classifier with 800 decision trees.
- Logistic Regression with L1 Penalty (L1LR) [26]. This regression-based method performs both variable selection and classification. We used the same inputs as those given to the Random Forest model and conducted 10-fold cross-validation to determine the hyperparameters.
- BiLSTM [27]. This is an RNN that performs well with text classification tasks and is used for sentiment analysis and question classification. The text encoder is a two-layer BiLSTM, whose output hidden states are concatenated with the journal feature representations, which use simple fully connected layers. The word embedding dimensions and BiLSTM hidden states were 128 and 256, respectively, and we applied a fully connected layer and a SoftMax function to make the final prediction.
- BiLSTM + Attention [28]. This is the same BiLSTM with an additional attention layer at the text encoder output.
- TextCNN [29]. This CNN is used for sentence classification tasks and has a kernel size of 32, which is used to extract sentence-level features. For journal feature representations, we applied the BiLSTM method and fed the concatenated representations into the classifier.
- TextRCNN [30]. This combination CNN + RNN uses the BiLSTM architecture for the RNN. The BiLSTM’s output is concatenated with the text embeddings, and a global max pooling layer is applied to obtain the final text representation. The text and journal representations are concatenated and used for the final prediction.
- PubMedBERT. In this method, PubMedBERT is used as text encoder and then fine-tuned during the training process. Then, the title, abstract, and journal features represented by feature encoders are directly concatenated for final classification.

Our dataset was randomly split into training (80%) and testing (20%). After training each on the same training dataset, we evaluated their prediction performances on the testing dataset. We counted the true positives (TPs), which reflect number of correctly predicted positive values, true negatives (TNs), which denote the number of correctly predicted negative values, false positives (FPs), which denote number of samples incorrectly predicted as positive, and false negatives (FNs), which denote the number of samples incorrectly predicted as negative. Based on these, Precision = TP / (TP + FP), Recall = TP / (TP + FN), Specificity = TN / (FP + TN), Accuracy = (TP + TN) / (TP + FP + FN + TN), F1-score = 2 × [(Precision × Recall) / (Recall + Precision)], and area under the receiver operating characteristic curve (AUC) = ${\int }_{0}^{1}Recall \text{d}\left(Precision\right).$ Finally, to illustrate the computational complexity of PAJO, we compare it with other deep learning baselines using FLOPs(T), i.e., the floating point operations per second (Unit is T).

### Data exploration and illustration

**Fig. 4:**
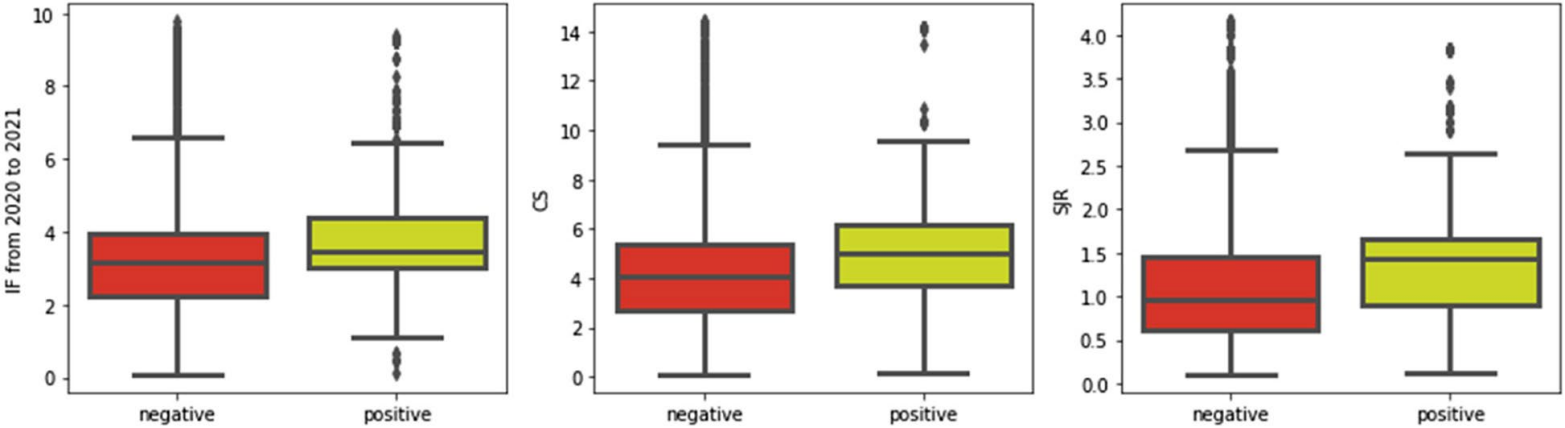
Boxplots of Journal Impact Factor (IF) from 2020 to 2021, CiteScore (CS), and Scientific Journal Ranking (SJR) reflecting positive vs. negative samples. Positive samples have higher journal feature values than negative samples, indicating that these features are useful in distinguishing high-quality articles

Prior to applying PAJO, we conducted a text and journal feature data exploration for illustrative purposes, which is useful when envisioning the state space and expected model outcomes. To get an idea of the influence of journal features on classification performance, we compared the distributions of Sets B and C in terms of IF, CS, and SJR. The representative boxplots are shown in Fig. 4. As can be seen, the values of IF, CS, and SJR in the positive samples were significantly higher than those in the negative samples. This finding suggests that journal features are usually positively correlated with article quality.

**Fig. 5:**
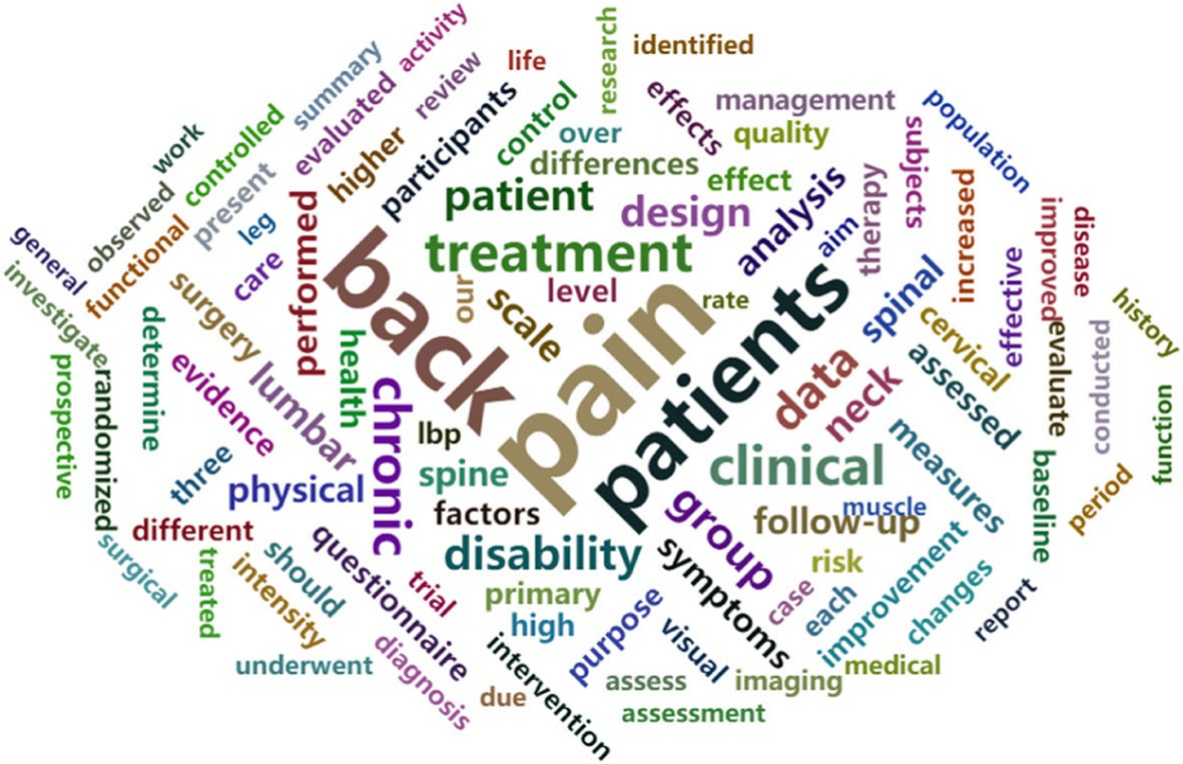
Word cloud containing the top 100 most frequent words found in the full sample set

Next, we focus on the text features extracted from titles and abstracts. To visualize these features, we calculated the frequency of each word from Set A and present the top 100 with the highest frequencies as a word cloud in Fig. 5. The higher the frequency, the larger the word. The highest-frequency words are intuitively related to neck pain based on the original search criteria. This demonstrates that our dataset is suitable for screening new articles.

### Experimental results

Table 1 lists the predicted performance values of each model using the same test dataset. As shown, our PAJO model achieved the best prediction performance. PAJO surpassed the strongest baseline by 0.75% in Accuracy, 1.91% in F1-score, and 2.25% in AUC. These results demonstrate the improved predictive ability of this model. However, when evaluated based on precision and specificity, L1LR achieved the best results. Notably, Precision and Recall are trade-off measures, meaning that they should be assessed together. Recall can be more important than Precision as a higher recall value can avoid missing important articles [5]. Thus, although PAJO does not achieve the best precision, its good recall performance indicates a higher practical applicability. As suggested by Recall, the phenomenon of false negatives is noteworthy. To avoid false negatives, a practical screening procedure can be used. Specifically, we can first set a lower threshold to allow more samples to be counted as positive. We hope that more true positive samples can be covered in this manner. Then all the predicted positive samples are sorted in a decreasing order of their predicted probabilities, which are easier for researchers to screen. Finally, focusing on the FLOPs(T), we find our proposed PAJO indeed has a bigger computational complexity than other deep leaning models. This is mainly because we include PubMedBERT and also use the attention mechanism. This architecture of PAJO contributes to its superior performance in classification.

**Table 1:** Experimental results of different models when evaluated by Precision, Recall, Specificity, Accuracy, F1 Score, FLOPs, and area under the receiver operating characteristic curve (AUC)

Model	Precision	Recall	Specificity	Accuracy	F1-score	AUC	FLOPs(T)
Random Forest	59.11	79.10	76.54	77.31	67.66	85.28	
L1LR	**75.00**	52.24	**92.54**	80.45	61.58	85.64	
BiLSTM	64.86	59.70	86.14	78.21	62.18	82.21	0.072
BiLSTM + Attention	66.67	68.66	85.29	80.30	67.65	83.63	0.080
TextCNN	66.67	61.69	86.78	79.25	64.08	85.22	0.0001
TextRCNN	61.54	79.60	78.68	78.96	69.41	86.15	0.077
PubMedBERT	71.69	78.11	86.78	84.18	74.76	89.59	3.047
PAJO	71.55	**82.59**	85.92	**84.93**	**76.67**	**91.84**	15.236

*Definitions *— *BiLSTM *Bidirectional long short-term memory, *L1LR *Logistic regression with L1 penalty, *PAJO *Paper title, Abstract, and Journal model, *TextCNN *Text-based convolutional neural network, *TextRCNN *Text-based recurrent convolutional neural network, *PubMedBERT *the fine-tuned PubMedBERT model

## Discussion

### Ablation experiments

As discussed in Methods section, the PAJO model consists of a text encoder, an attention encoder, and a journal feature encoder. To exploit textual information, both the title and abstract of an article are used. We conducted a series of ablation experiments to explore the utility of each part, as it contributes to the performance of the whole. Specifically, we considered five scenarios based on the intake of three types of features: title (T), abstract (A), and journal (J). Hence, PAJO-T intakes only title features, and PAJO-A intakes only article features, both into the text encoder. PAJO-TJ intakes titles into the text encoder and uses the journal feature encoder to extract journal information. Similarly, PAJO-TA intakes article titles and abstracts into the text encoder. PAJO-AJ intakes article abstracts into the text encoder and uses the journal feature encoder to extract journal information. Lastly, PAJO-FULL refers to a fully functional model. This conclusion is consistent with that of a study by Zhang et al., who discovered that their BERT-CAM model, which also utilizes abstract characteristics, performed better than other techniques in terms of accuracy, precision, recall, and F1 value [31]. This shows that an important aspect of the effectiveness of NLP models is their utilization of abstract properties.

Table 2 lists the detailed results of the ablation experiments. Notably, PAJO-FULL achieved the best performance, apart from Precision. Interestingly, PAJO-A had the highest precision, 72.22%, whereas PAJO-FULL only scored 71.55%. However, as discussed, Precision and Recall are defined according to a given threshold, and they should not be interpreted as performance measures alone. A more integrated measure for Precision and Recall is the AUC and we found PAJO-FULL has the highest AUC value. Based on Table 2, we also found that abstract features play a significant role in overall performance, as their removal resulted in much lower scores in all metrics. The largest drop was observed in precision, which decreased 12.68% from 71.55% (PAJO-FULL) to 58.87% (PAJO-TJ).

**Table 2:** Results of ablation experiments when evaluated on Precision, Recall, Specificity, Accuracy, F1 score, and area under the response operating characteristic curve (AUC)

Model	Precision	Recall	Specificity	F1-score	Accuracy	AUC
PAJO-T	69.19	72.64	86.14	70.87	82.09	86.71
PAJO-A	**72.22**	71.14	88.27	71.68	83.13	89.72
PAJO-TJ	58.87	77.61	76.76	66.95	77.01	86.98
PAJO-TA	70.40	78.11	85.93	74.06	83.58	89.15
PAJO-AJ	71.95	79.10	**86.78**	75.36	84.48	89.98
PAJO-FULL	71.55	**82.59**	85.92	**76.67**	**84.93**	**91.84**

PAJO-FULL performed best in all metrics apart from Precision. *Definitions*: — *PAJO *Paper title, Abstract, and JOurnal model, *PAJO-A *Base model only intakes abstract features, *PAJO-AJ *Base model only intakes abstract and journal features, *PAJO-FULL *Complete model, *PAJO-T *Base model only intakes title features, *PAJO-TJ *Base model only intakes title and journal features, *PAJO-TA *Base model only intakes abstract and title features

### PAJO prediction performance

To further evaluate the efficacy of the proposed model in screening important articles related to neck pain, we additionally collected articles published in 2022. We regarded these articles as a new testing set, and then applied PAJO to classify them with prediction probabilities. The higher the probability, the more important the article. The prediction threshold was set to 0.5, which resulted in 60 positively classified studies. For comparison purposes, we randomly selected the same number of articles with prediction probabilities larger than 0.5 published in 2021.

All articles were evaluated from two perspectives by a trained radiologist who was blinded to the prediction results. The first perspective was the *degree of relationship* to the topic, neck pain, rated on a scale of 1–4. The higher the score, the stronger the relationship. The second was article *quality*, again rated on a scale of 1–4. This scale is based on the Grading of Recommendations Assessment, Development, and Evaluation method used by the American College of Radiology Appropriateness Criteria 1, 2. Finally, the two scores were simply summed up. The summation has a scale of 2–8 for each article.

**Fig. 6:**
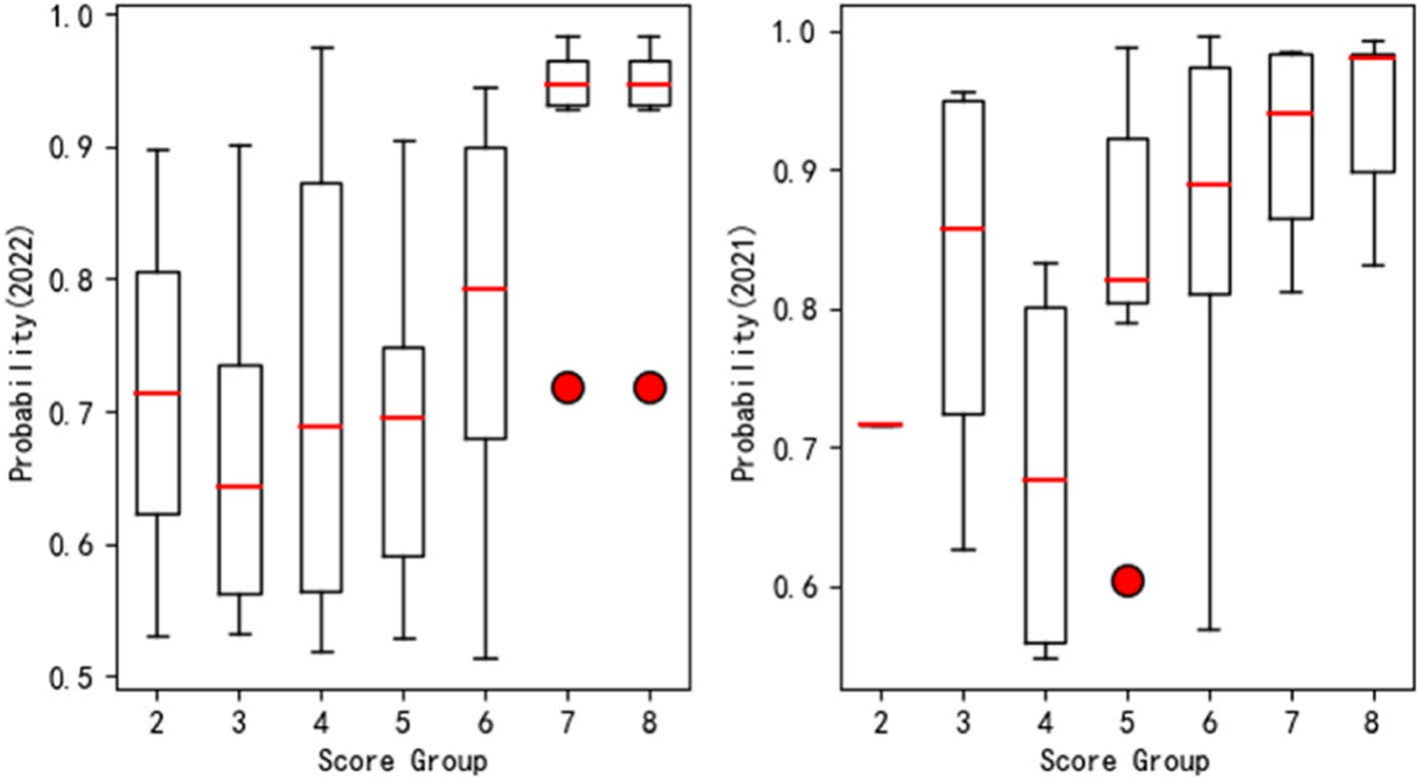
Boxplots of article applicability + quality. The y-axis reflects the interquartile model predictions, where the red dots are outliers. The x-axis reflects the scale of expert-provided ground-truth applicability + quality, with “8” being the highest. The left panel reflects articles published in 2022, and the right panel reflects articles published in 2021

We examined the prediction distributions, which are illustrated as boxplots in Fig. 6. As shown, articles in groups with higher expert scores had correspondingly higher prediction scores. For example, the median probabilities of articles falling into the score groups of seven and eight are all around 0.95; while the median probabilities of articles falling into the other groups are all below 0.9. This trend indicates good consistency between the model predictions and the ground truth. For each score group, we also tested the differences in predictions made for articles published in 2022 and 2021. The results showed no significant differences between the groups, suggesting that articles published in different years have similar patterns. The PAJO model’s ability to handle large-scale datasets and its robustness to noise suggest that it could be used in a variety of real-world applications, such as information retrieval, document classification, and recommendation systems. Future studies could examine these prospective uses and assess how well the model functions in them. Future research may further examine the PAJO model’s application in fields other than neck pain research to gauge its adaptability and versatility.

The PAJO model might benefit from the incorporation of more varied features, based on the findings of the ablation experiments. For instance, the performance of the PAJO model might be enhanced using pre-trained language models (BERT) in the BERT-CAM [31] and AFR-BERT by Ji et al. [32] models. Additionally, the Bidirectional Long Short-Term Memory (BiLSTM) used by the AFR-BERT model for pre-processing data might be considered for incorporation into the PAJO model.

## Conclusions

CPGs are important documents that provide healthcare best practices for clinicians, administrators, the public, and program managers. The screening of high-quality related articles plays a vital role in the development of CPGs. However, given the vast number of articles, manual curation is too time-consuming and labor-intensive. To help resolve this problem, we developed the PAJO model to assist practitioners in screening high-quality articles automatically. This model includes text, attention, and journal feature encoders. In addition to titles and abstracts, the model comprehensively considers article characteristics (e.g., IF and SJR). Taking “neck pain” as the focus area of this study, we constructed a dataset with highly relevant articles extracted via a query from PubMed. We then conducted extensive experiments using PAJO to identify the most important articles. Our results show that PAJO model performs better than several state-of-the-art methods on the literature screening task. To further verify the model efficacy, we tested its prediction performance using articles published in 2022 as the test set. Experts volunteered to provide ground-truth evaluations. The results show that there was a strong matching between model predictions and ground-truth results in terms of identifying the highest-quality articles. This result shows that the PAJO model can assist CPG curators with their jobs.

We now present some limitations of the PAJO model, which may set future directions. Treating all articles not cited by CPGs as negative samples is a crude first approximation. A more flexible approach using positive unlabeled learning may be applied to handle these negative samples. Second, the feature extraction method in PAJO may bias the model towards certain journals. Further investigation in this regard is needed. Mining textual characteristics from articles is another direction for future work. The prediction performance of PAJO was evaluated by a single trained radiologist. More evaluations by other radiologists must be conducted in future. Here, we examined the performance of PAJO on neck pain. In the future, the PAJO model can be extended to more diseases to verify its wide applicability.

## Data Availability

The datasets and codes used during the current study are available at https://github.com/xh621/PAJO-Deep-Learning-Model.

## Abbreviations

AUC: Area under the receiver operating characteristic curve
BERT: Bidirectional encoder representations from transformers
BiLSTM: Bidirectional long short-term memory
CNN: Convolutional neural network
CPG: Clinical practice guideline
CS: CiteScore metric
FN: False negative
FP: False positive
IF: Journal impact factor
L1LR: Logistic Regression with L1 Penalty
LSTM: Long short-term memory
NLP: Natural language processing
PAJO: Our proposed Paper title, Abstract, and JOurnal deep-learning model
ReLU: Rectified linear unit
RNN: Recurrent neural network
SJR: Scientific journal ranking
SNIP: Source-normalized impact per paper
SVM: Support vector machine
TN: True negative
TP: True positive

## Footnotes

1
https://pubmed.ncbi.nlm.nih.gov/.

## References

[1] Chen Y, Yang K, Marušić A, Qaseem A, Meerpohl JJ, Flottorp S (2017). A reporting tool for practice guidelines in health care: the RIGHT statement. Ann Intern Med.

[2] Shekelle PG (2018). Clinical practice guidelines: what’s Next?. J Am Med Assoc.

[3] Fire M, Guestrin C (2019). Over-optimization of academic publishing metrics: observing Goodhart’s Law in action. GigaScience.

[4] Harmsen W, de Groot J, Harkema A, van Dusseldorp I, De Bruin J, Van den Brand S et al. Artificial intelligence supports literature screening in medical guideline development: Towards up-to-date medical guidelines. Medicine. 2021. 10.5281/ZENODO.5031907.

[5] Feng Y, Liang S, Zhang Y, Chen S, Wang Q, Huang T (2022). Automated medical literature screening using artificial intelligence: a systematic review and meta-analysis. J Am Med Inform Assoc.

[6] Dessi D, Helaoui R, Kumar V et al. TF-IDF vs word embeddings for morbidity identification in clinical notes: an initial study. 2021;DOI 10.5281/zenodo.4777594.

[7] Kumar V, Recupero DR, Riboni D (2020). Ensembling classical machine learning and deep learning approaches for morbidity identification from clinical notes. IEEE Access.

[8] Wu S, Roberts K, Datta S, Du J, Ji Z, Si Y (2020). Deep learning in clinical natural language processing: a methodical review. J Am Med Inform Assoc.

[9] Mullenbach J, Wiegreffe S, Duke J, Sun J, Eisenstein J. Explainable prediction of medical codes from clinical text. 2018; DOI:10.18653/v1/N18-1100.

[10] Prabhakar SK, Won DO (2021). Medical text classification using hybrid deep learning models with multihead attention. Comput Intell Neurosci.

[11] Zhang Y, Liang S, Feng Y, Wang Q, Sun F, Chen S (2022). Automation of literature screening using machine learning in medical evidence synthesis: a diagnostic test accuracy systematic review protocol. Syst Rev.

[12] Lan Z, Chen M, Goodman S, Gimpel K, Sharma P, Soricut R. ALBERT: A lite BERT for self-supervised learning of language representations. In International Conference on Learning Representations. 2020:1311–28.

[13] Beltagy I, Lo K, Cohan A. SciBERT: A Pretrained Language Model for Scientific Text. In Proceedings of the 2019 Conference on Empirical Methods in Natural Language Processing and the 9th International Joint Conference on Natural Language Processing. 2019:3615–20.

[14] Lee J, Yoon W, Kim S, Kim D, Kim S, So CH (2020). BioBERT: a pre-trained biomedical language representation model for biomedical text mining. Bioinformatics.

[15] Moen H, Alhuwail D, Björne J, et al. Towards Automated Screening of Literature on Artificial Intelligence in Nursing. Stud Health Technol Inform. 2022;290:637–40. 10.3233/SHTI220155 35673094

[16] Kumar V, Recupero DR, Helaoui R (2022). K-LM: knowledge augmenting in Language Models within the Scholarly Domain. IEEE Access.

[17] Lin TY, Goyal P, Girshick R, He K, Dollar P (2020). Focal loss for dense object detection. IEEE Trans Pattern Anal Mach Intell.

[18] Gu Y, Tinn R, Cheng H, et al. Domain-specific language model pretraining for biomedical natural language processing. ACM Trans Comput Healthc. 2021;3(1):1–23.

[19] Garfield E (2006). The history and meaning of the journal impact factor. J Am Med Assoc.

[20] Van Noorden R (2016). Impact factor gets heavyweight rival. J Cit Rep.

[21] Falagas ME, Kouranos VD, Arencibia-Jorge R, Karageorgopoulos DE (2008). Comparison of SCImago journal rank indicator with journal impact factor. FASEB J.

[22] Leydesdorff L, Opthof T (2010). Scopus’s source normalized impact per paper (SNIP) versus a journal impact factor based on fractional counting of citations. J Am Soc Inf Sci.

[23] Roldan-Valadez E, Salazar-Ruiz SY, Ibarra-Contreras R, Rios C (2019). Current concepts on bibliometrics: a brief review about impact factor, eigenfactor score, CiteScore, SCImago journal rank, source-normalised impact per paper, H-index, and alternative metrics. Ir J Med Sci.

[24] Devlin J, Chang MW, Lee K, Toutanova K, Bert. Pre-training of deep bidirectional transformers for language understanding. In Annual Conference of the North American Chapter of the Association for Computational Linguistics. 2019:4171–86.

[25] Sun Y, Li Y, Zeng Q, et al. Application research of text classification based on random forest algorithm. In 2020 3rd International Conference on Advanced Electronic Materials, Computers and Software Engineering (AEMCSE). 2020:370–4.

[26] Aseervatham S, Antoniadis A, Gaussier E, Burlet M, Denneulin Y (2011). A sparse version of the ridge logistic regression for large-scale text categorization. Pattern Recognit Lett.

[27] Qing L, Linhong W, Xuehai D (2019). A novel neural network-based method for medical text classification. Future Internet.

[28] Deng J, Cheng L, Wang Z (2021). Attention-based BiLSTM fused CNN with gating mechanism model for chinese long text classification. Comput Speech Lang.

[29] Kim Y. Convolutional neural networks for sentence classification. In Proceedings of the 2014 Conference on Empirical Methods in Natural Language Processing (EMNLP). 2014:1746–51. 10.18653/v1/d16-1076 PMC5300751 28191551

[30] Lai S, Xu L, Liu K, et al. Recurrent convolutional neural networks for text classification. In The 29th AAAI Conference on Artificial Intelligence. 2015:2267–73.

[31] Pan L, Lim WH, Gan Y (2023). A method of Sustainable Development for three Chinese short-text datasets based on BERT-CAM. Electronics.

[32] Mingyu J, Jiawei Z, Ning W (2022). AFR-BERT: attention-based mechanism feature relevance fusion multimodal sentiment analysis model. PLoS ONE.

